# CAR-T Cell Therapy for HIV Cure

**DOI:** 10.3390/v15091793

**Published:** 2023-08-23

**Authors:** Maria Salgado

**Affiliations:** 1 AIDS Research Institute, IrsiCaixa, 08916 Barcelona, Spain; msalgado@irsicaixa.es; 2 Institute for Health Science Research Germans Trias i Pujol (IGTP), 08916 Barcelona, Spain; 3 CIBERINFEC, Carlos III Health Institute (ISCIII), 28220 Madrid, Spain

The HIV-1 latent reservoir is considered the major barrier to achieve the eradication, although some evidences indicate that curing HIV-1 is a feasible goal. Different immunotherapy-based strategies have been assayed to be able to kill HIV-infected cells. Therapeutic vaccination has obtained interesting results in terms of increasing HIV-1-specific immune responses, with a limited reduction in HIV reservoirs. Additionally, inhibitory receptors, also called immune checkpoint inhibitors (ICIs), can exert their influence on T-cell responses but with marginal results so far. To date, the chimeric antigen receptor (CAR) T cell therapy postulates as the most suitable strategy in enhancing the T cell responses to levels that can mimic the alloreactivity of the allo-HSCT.

Previous investigations have focused on second-generation CAR-T cells carrying the CD4 binding site as an extracellular domain since this part of the CD4 molecule has a high affinity for gp120 HIV-1 proteins. The rationale is to target cells that express this viral protein on their surface. Although some optimistic results have been obtained, this approach has the disadvantage that the CD4 molecule on the surface of the CD8+ T cells makes them susceptible to HIV-1 infection. For that reason, the new generation of CARs has included broadly neutralizing antibodies (bNAbs) as an additional domain to target HIV-1. Since this is a field undergoing significant development, this Special Issue of *Viruses* aims to explore new advancements in CAR-T cell therapies as immunotherapies to cure HIV. 

Primary research and review articles pertaining to CAR-T cell development as an immunotherapy to study and eliminate HIV latency have been published concerning this issue. 

We would like to thank all of the authors for their submissions and the great work expanding our knowledge of CAR-T cells in the context of viral elimination and latency eradication. We learned about the development of HIV-resistant CAR-T Cells via CRISPR/Cas-Mediated CAR integration into the CCR5 Locus in an interesting article from Frederik Holm Rothemejer et al. [1] (https://doi.org/10.3390/v15010202). We have also seen the related cytotoxic dynamics that are associated with immunotherapies in Barun Majumder et al. (https://doi.org/10.3390/v15071454) [2].

In addition, we have published thorough reviews, first highlighting the history and challenges for the CAR-T cell therapy development in HIV (Gerard Campos-Gonzalez et al., https://doi.org/10.3390/v15030789) [3]. The second review highlights the technical challenges in the quantification of HIV-1-infected and CAR-T cells in the setting of lentiviral CAR gene delivery as well as the challenges in the identification of cells expressing target antigens (Amanda M. Buck et al., https://doi.org/10.3390/v15051126 [4]).

Finally, as a guest editor, I would like to conclude that CAR-T-cell-based immunotherapies are generating promising and original approaches with encouraging results, and it may be that a combination of new approaches will be crucial to overcoming the difficulties in achieving an HIV cure.
